# Supplementary material for: Seasonality of downward carbon export in the Pacific Southern Ocean revealed by multi-year robotic observations
Source: Nat Commun. 2023 Mar 8;14:1278. doi: 10.1038/s41467-023-36954-7 (PMC9995333; doi:10.1038/s41467-023-36954-7)
Supplement: Supplementary file 1 — Supplementary information [file 41467_2023_36954_MOESM1_ESM.pdf]

Supplementary information for

Seasonality of downward carbon export in the Pacific Southern Ocean revealed by multi-year robotic observations

Léo Lacour[1,2]*, Joan Llort[3], Nathan Briggs[4], Peter G. Strutton[1,5], Philip W. Boyd[1]

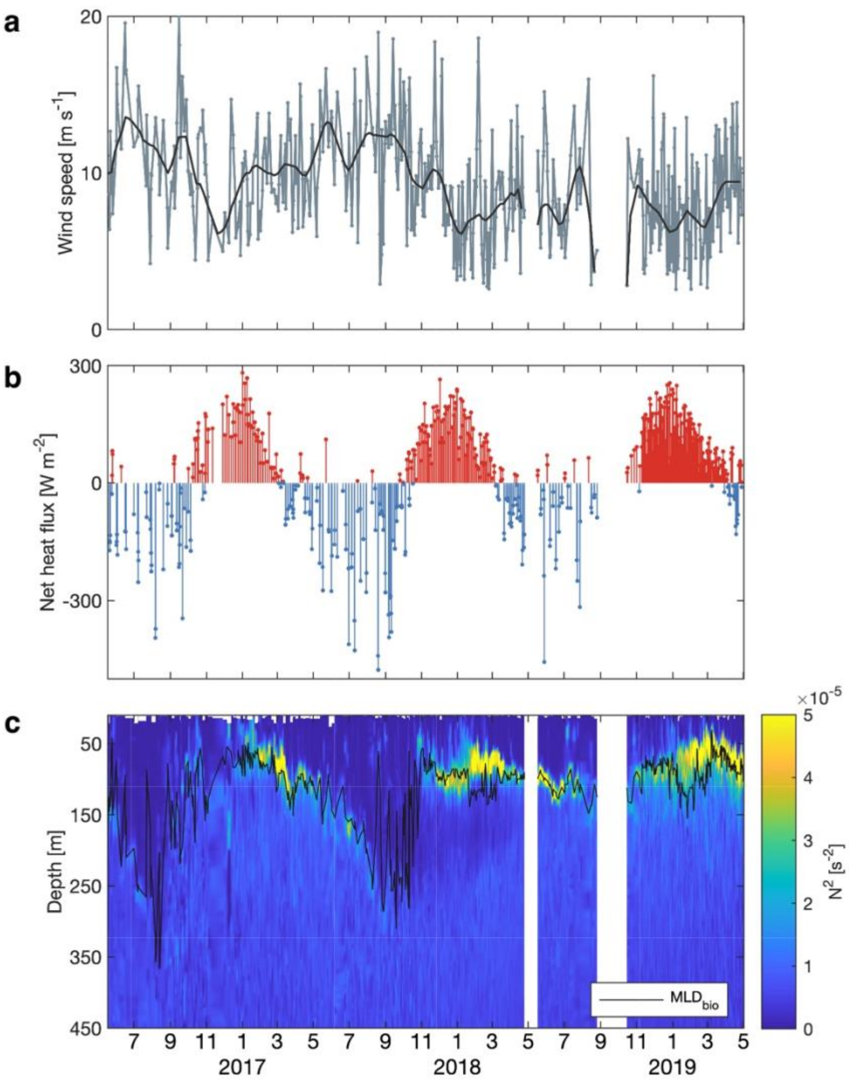

Figure S1 Environmental factors controlling phytoplankton phenology and vertical carbon export. (a) Daily wind speed at 10 m above the ocean surface, along the float trajectory. The black line is a 30-day smooth. **(b)** Daily net heat flux along the float trajectory, with positive values referring to a flux from the atmosphere to the ocean. Vertical sections of **(c)** Buoyancy frequency N^2.

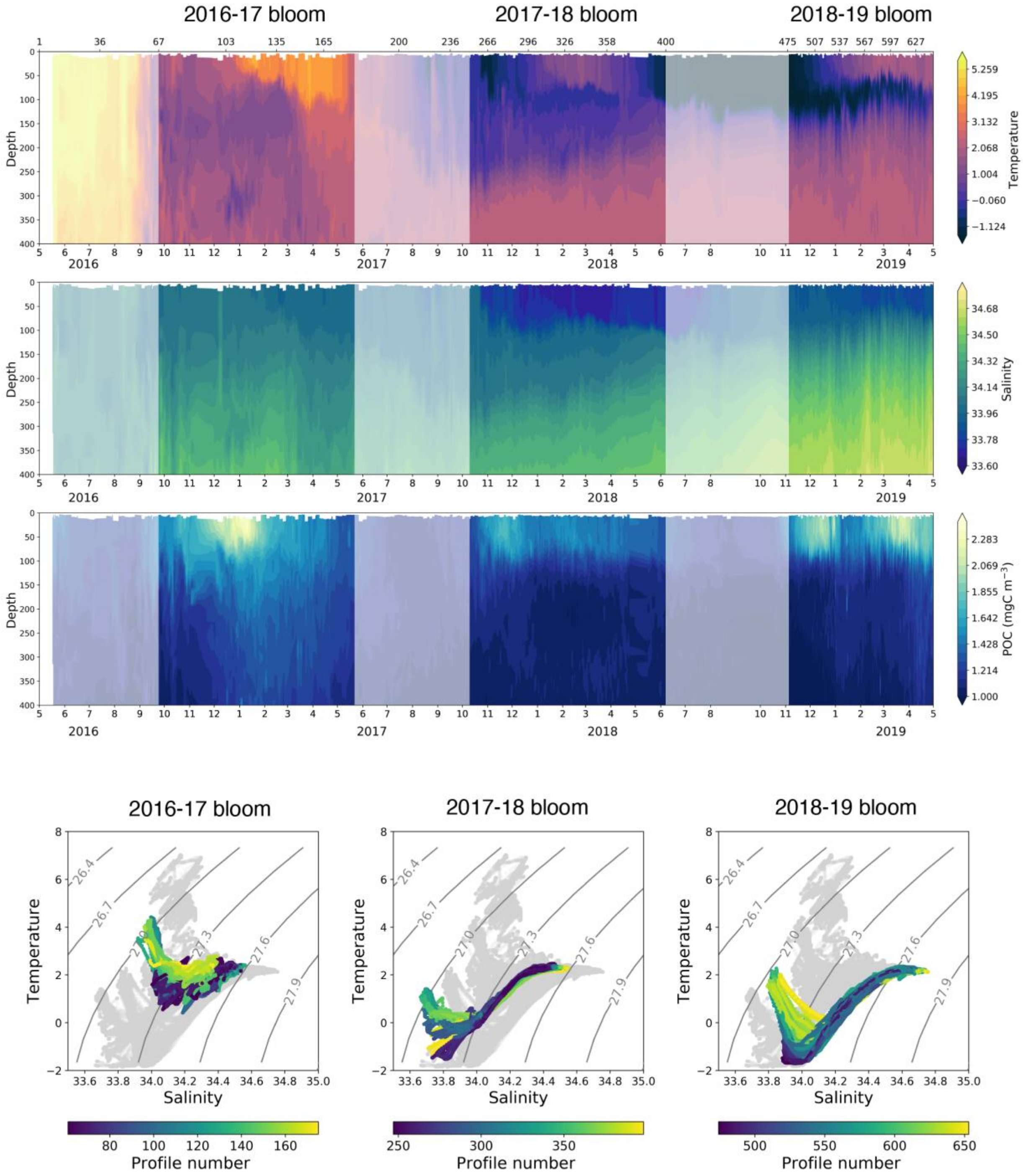

Figure S2 Pseudo-Lagrangian framework. The float timeseries was divided into three periods in which the contiguous nature of the water masses was verified (areas not shaded grey). The three TS diagrams at the bottom reveal that the float did not cross any strong water mass boundaries during each period of interest. Grey dots in the background show data points of the complete timeseries.

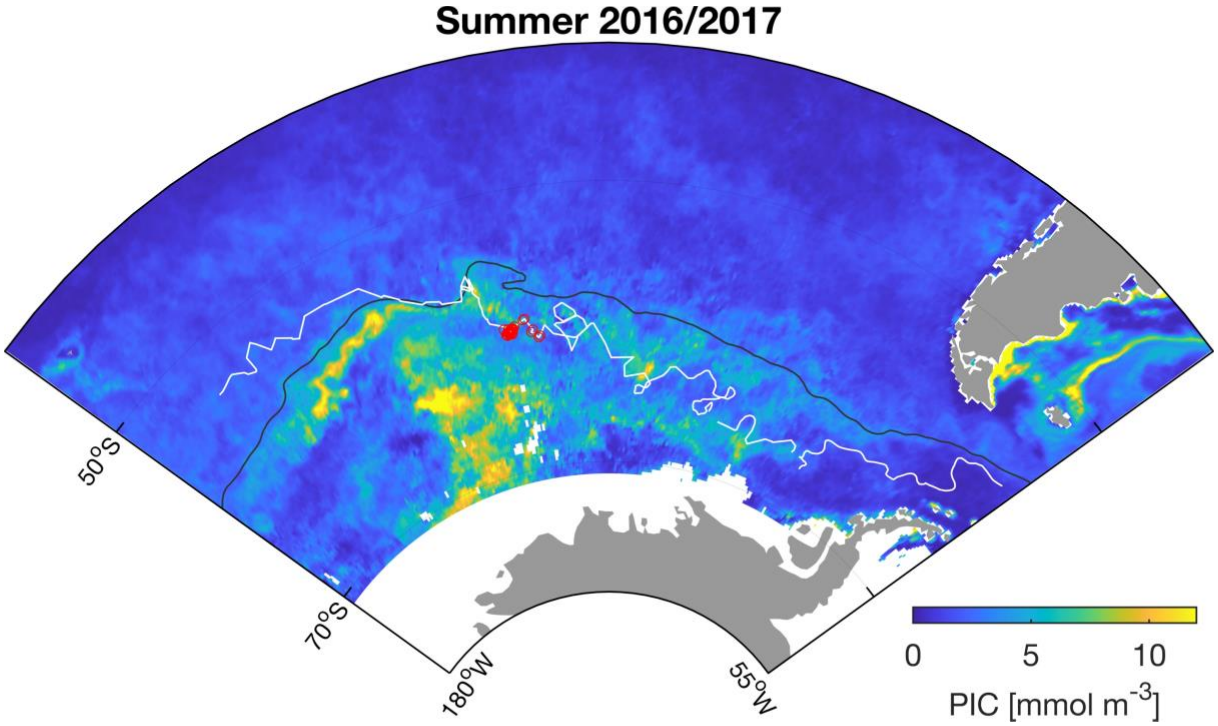

Figure S3 Satellite Particulate Inorganic Carbon (PIC) reveals the presence of a coccolithophore bloom in austral summer 2016-17. Trajectory of the float superimposed on summer (December 2016 to February 2017) composite GlobColour image (25 km) of PIC. Red circles indicate the location of the summer float profiles where we observed a strong decrease in the F_s / b_{bs} ratio.

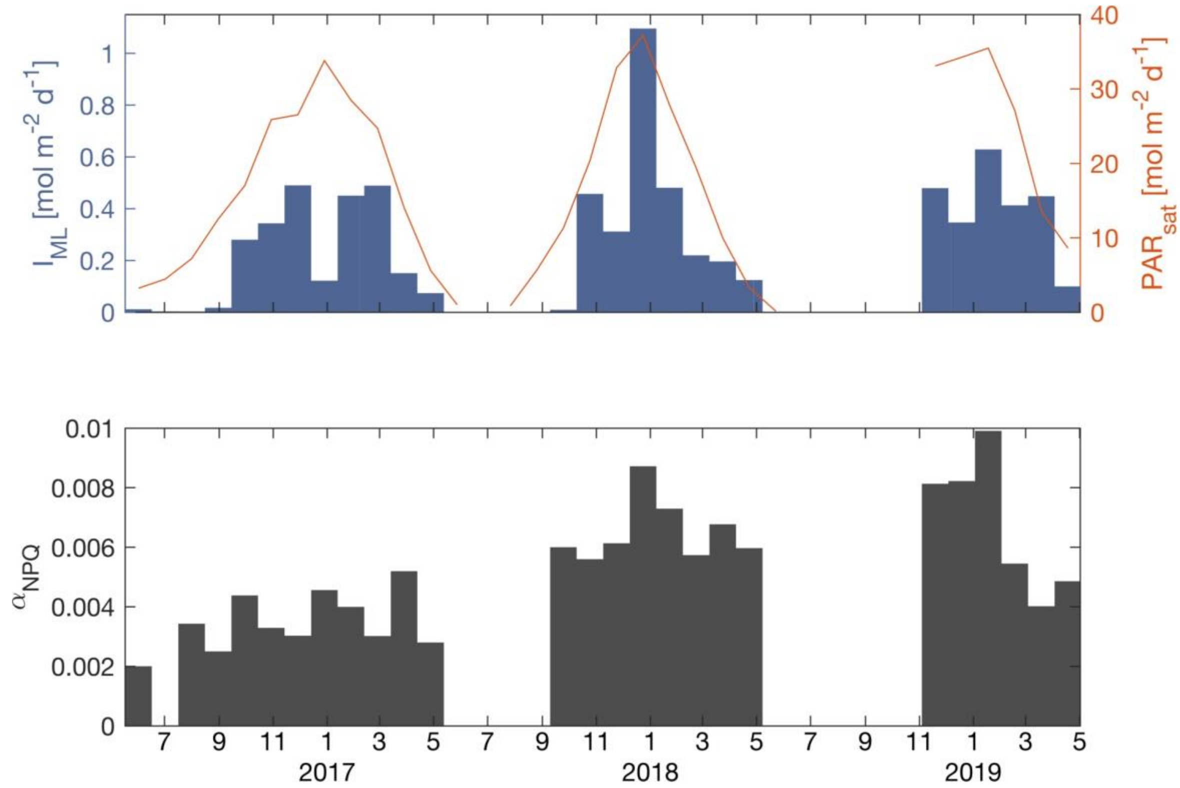

Figure S4 Iron and light limitation play a key role in shaping phytoplankton phenology in the SO. Top panel, median light level within the mixing layer (I_{ML}, blue bars) derived from satellite daily mean PAR (red curve). I_{ML} is an indicator of the light history of phytoplankton cells and has been previously used as a descriptor of photoacclimation[62]. Gaps in the timeseries are due to the winter polar night or the presence of sea ice. Bottom panel, initial slope of the non-photochemical quenching (NPQ) versus instantaneous iPAR curve (α_{NPQ}). α_{NPQ} is a proxy for phytoplankton iron limitation[36], the higher α_{NPQ}, the more iron-limited.

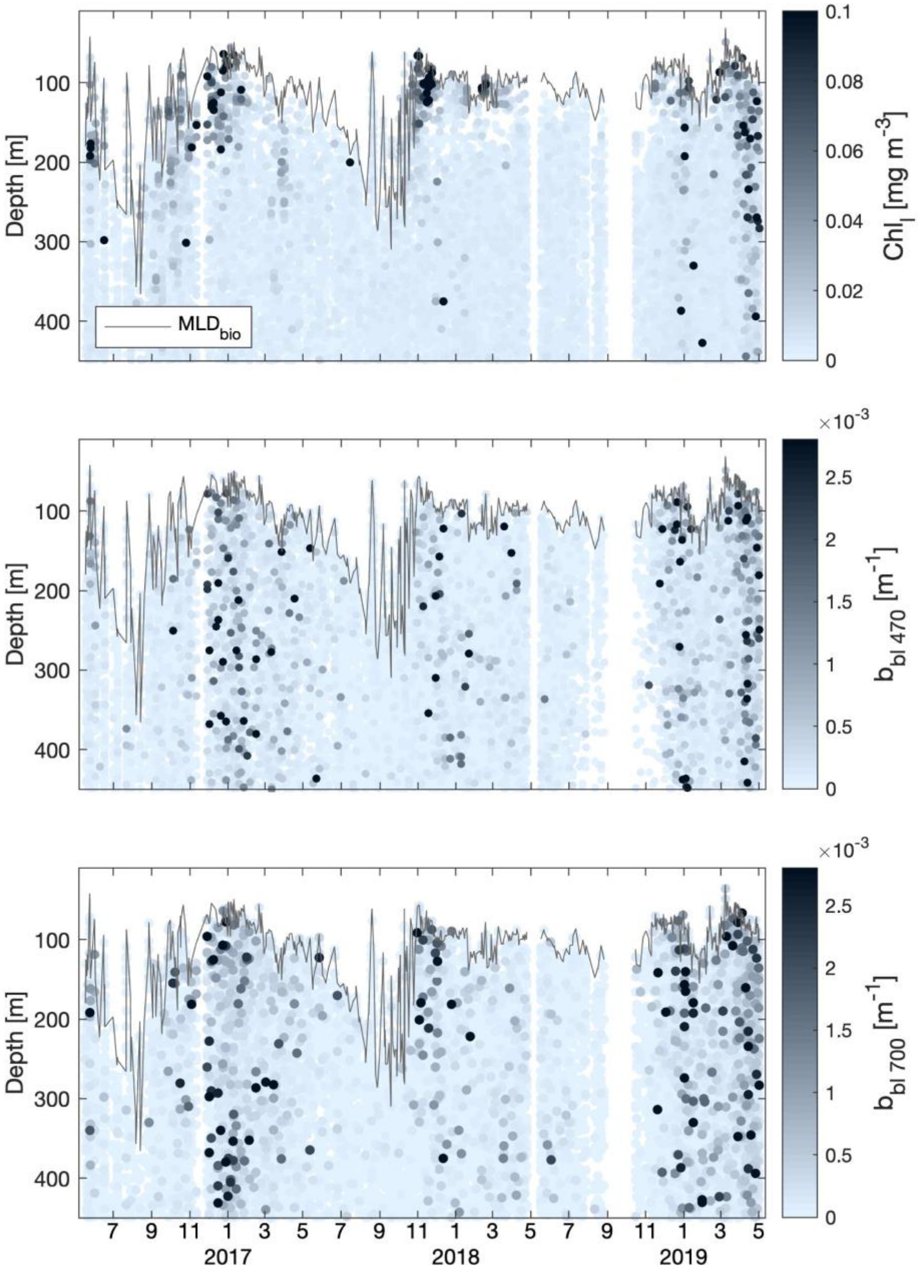

Figure S5 Optical spikes in the mesopelagic. Vertical profiles of large-particle fluorescence Chl_l (top), revealing the presence of fresh phytoplankton aggregates, and large-particle backscattering $b_{bl\ 470}$ (middle) and $b_{bl\ 700}$ (bottom), which additionally include fecal and detrital matter.

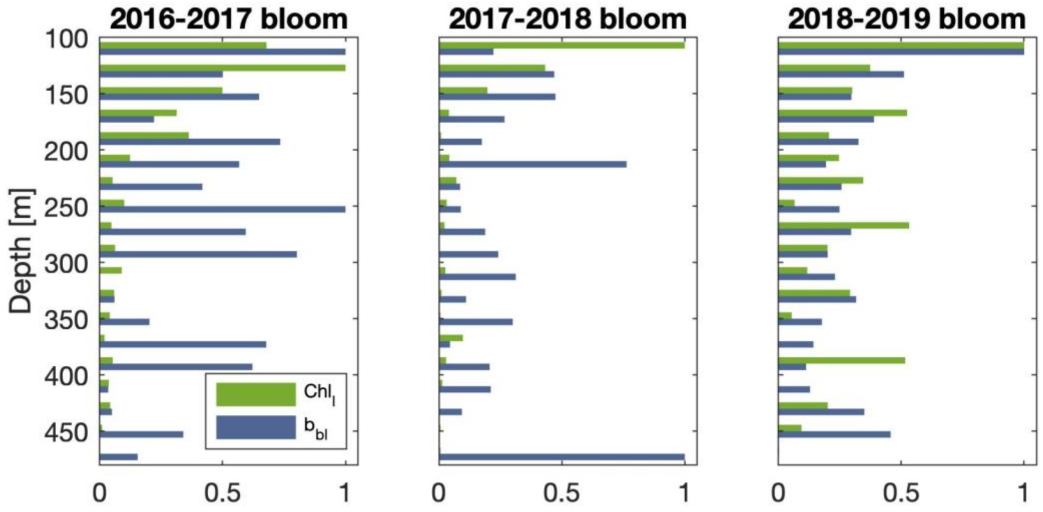

Figure S6 Vertical distribution of Chl$_l$ and b$_{bl}$, summed in 20 m bins over the course of the blooming period (from climax to 30 days after apex for the first two blooms and from climax to the end of the timeseries for the last bloom which shows two distinct POC peaks few months apart, see Fig. S2) and normalised by their minimum and maximum values. In contrast to b$_{bl}$, Chl$_l$ was quickly attenuated with depth, although this is less clear for the last 2018-19 bloom.

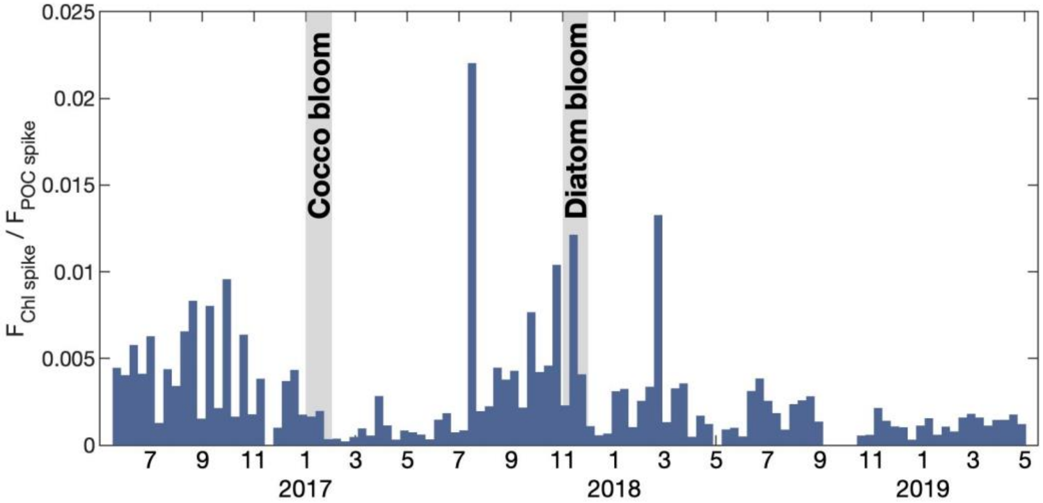

Figure S7 Freshness of the exported material in the upper mesopelagic. The gravitational flux derived from optical spikes in Chl ($F_{Chl\ spike}$) was normalised by the POC flux derived from b_{bp} spikes ($F_{POC\ spike}$) to estimate the freshness of the material in a 100 m bin below the mixing layer depth.

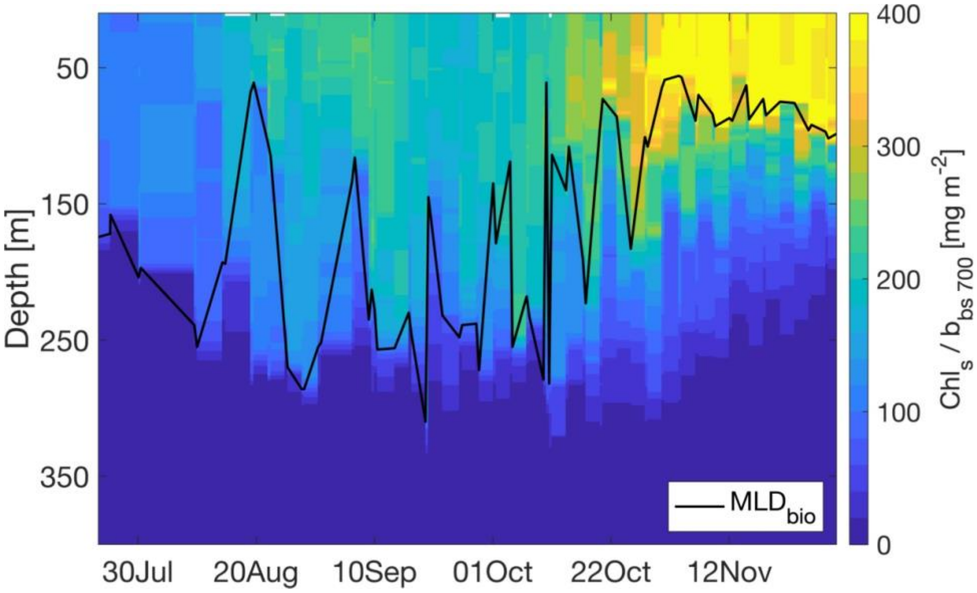

Figure S8 Illustration of the MLP during the 2017-18 bloom which shows how rapid and intermittent re-stratifications of the MLD isolates fresh particles at depth. The freshness of particles is revealed by the relatively high Chl_s / b_{bs} ratio (100-250 mg m^{-2}) observed below MLD_{bio}.

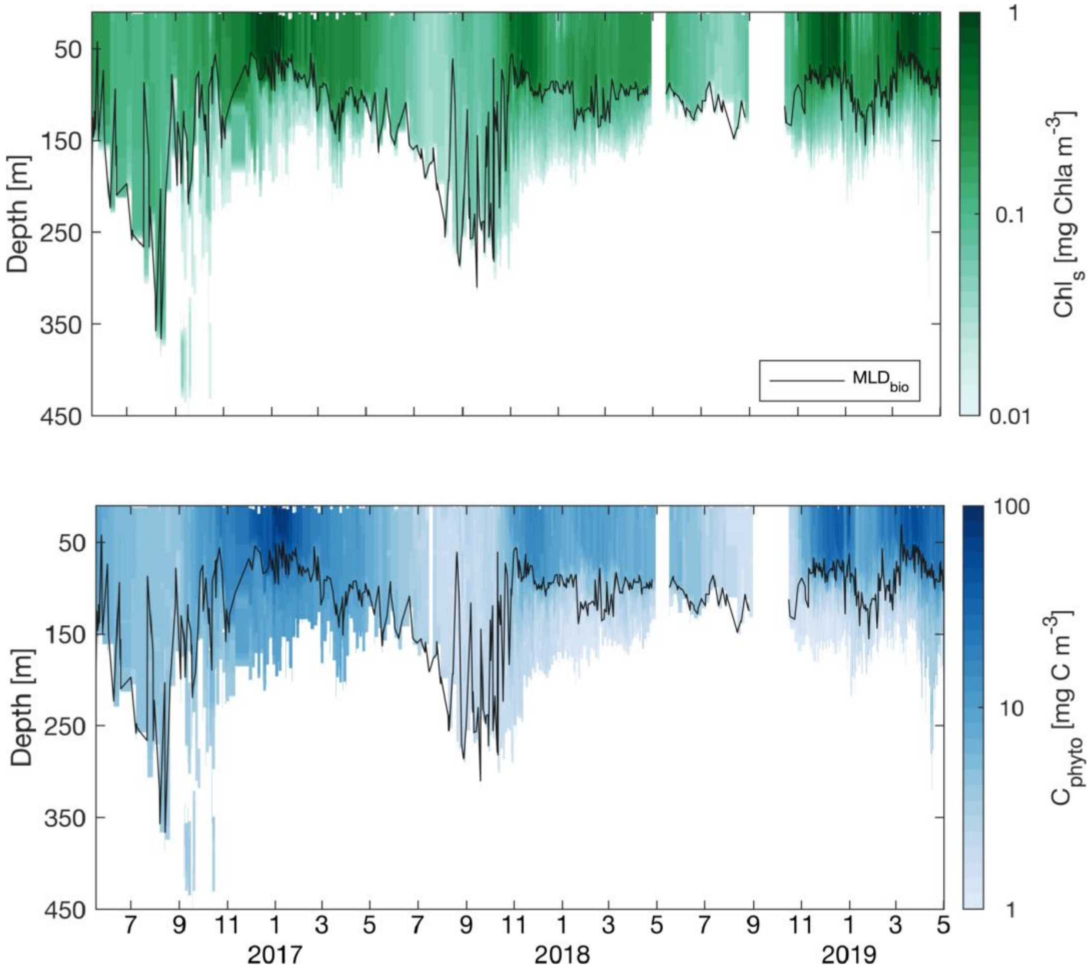

Figure S9 Phytoplankton biomass derived from optical measurements. Vertical profiles of small-particle Chlorophyll concentration (Chl_s, top), and phytoplankton carbon (C_{phyto}) derived from the backscattering at 470 nm ($b_{bp\ 470}$, bottom). Both colour bars are on a log scale. Black lines show the mixing layer depth. In the bottom panel, C_{phyto} was masked out where $Chl_s \leq 0$. Total phytoplankton carbon was then calculated by vertically integrating masked C_{phyto} over the whole water column, following Uchida et al. (2019)[68]. Note that SCMs are barely discernible in the top figure due to the use of running minimum and maximum filters to compute Chl_S, which partially erases subsurface peaks in fluorescence (see Methods).

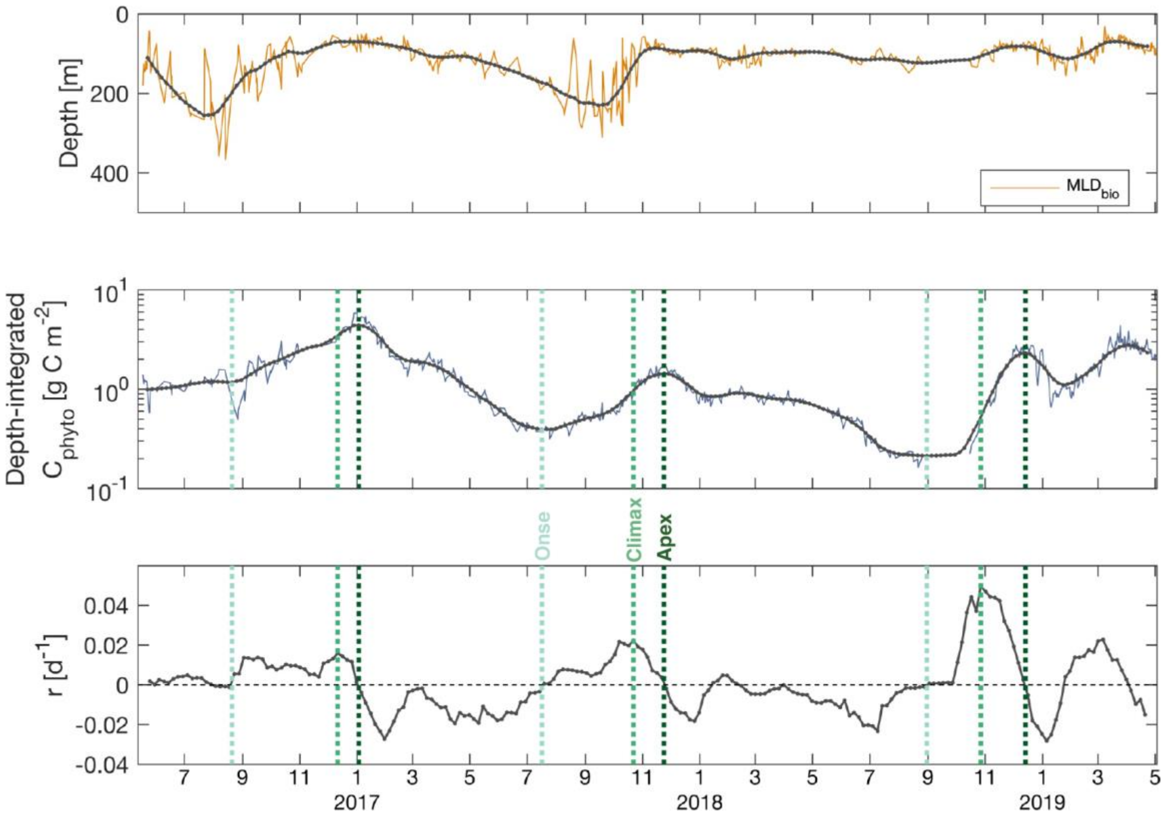

Figure S10 Phytoplankton phenology metrics. Top, mixing layer depth (MLD_{bio}). Middle, phytoplankton carbon. Continuous black lines in the top two panels show 30-day moving averages of these variables. Bottom, net growth rate r calculated from smoothed depth-integrated C_{phyto} (see Methods). Vertical dotted green lines indicate the timing of onset, climax and apex of the three phytoplankton blooms recorded by the float.

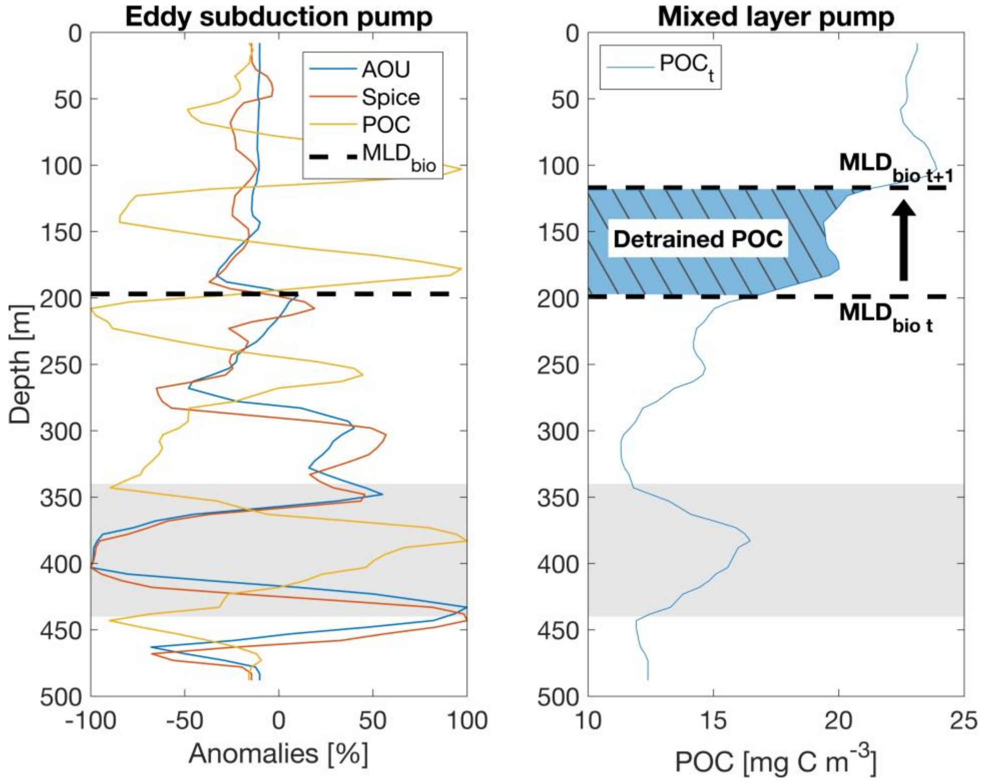

Figure S11 Profile example on 2016-09-07 to illustrate the subsurface signature of ESP export events (left panel) and the calculation of MLP flux (right panel). The shaded grey area in both panels highlights the layer defined by a potential density of 27.31 ± 0.04 kg m^{-3} where the ESP feature has been detected (co-located anomalies of AOU, spice and POC). The hatched light blue area highlights the remnant layer containing the POC detrained when MLD_{bio} shallowed between time t and t+1. In our study, all ESP features are much deeper than MLP remnant layers resulting in no double counting of physically-driven POC export by the two mechanisms (see also Fig. S14).

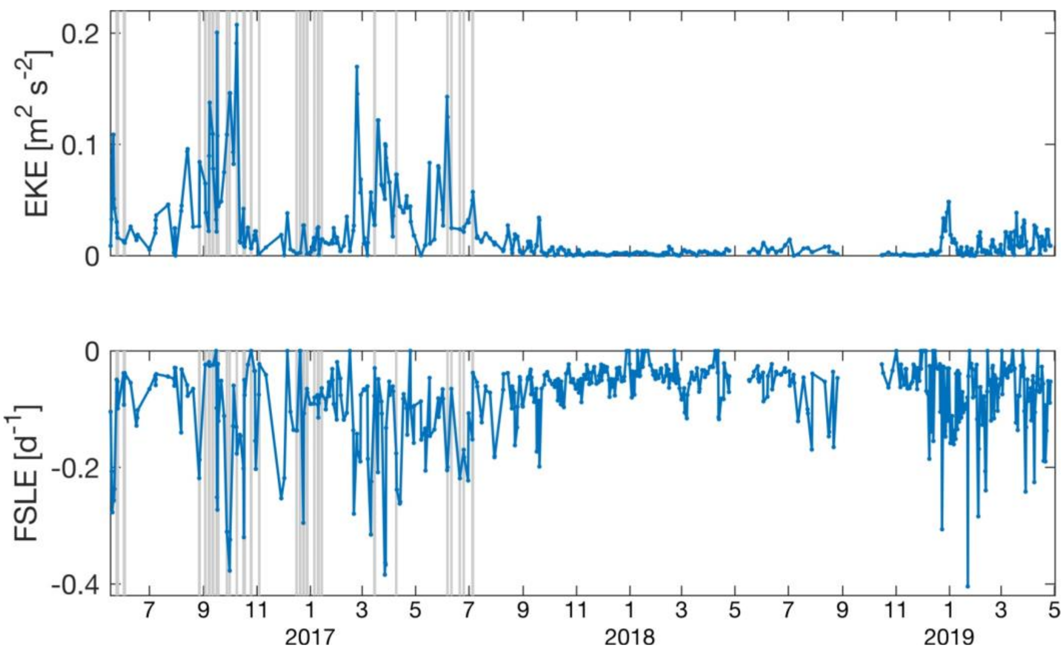

Figure S12 Satellite diagnostics of ESP events along the float trajectory. Top panel, eddy kinetic energy $EKE = 0.5\ (ugosa^2 + vgosa^2)$ where ugosa and vgosa are the zonal and meridian components of the geostrophic velocity anomalies, respectively, derived from multimission satellite altimetry gridded data (https://www.aviso.altimetry.fr/en/data/products/sea-surface-height-products/global.html). Bottom panel, backward-in-time Finite Size Lyapunov Exponents FSLE downloaded from aviso (https://www.aviso.altimetry.fr/en/data/products/value-added-products/fsle-finite-size-lyapunov-exponents.html). Large negative values indicate the presence of strong (sub)mesoscale fronts potentially associated with enhanced vertical velocities[74,75]. Vertical grey bars show the timing of ESP events detected along the float trajectory.

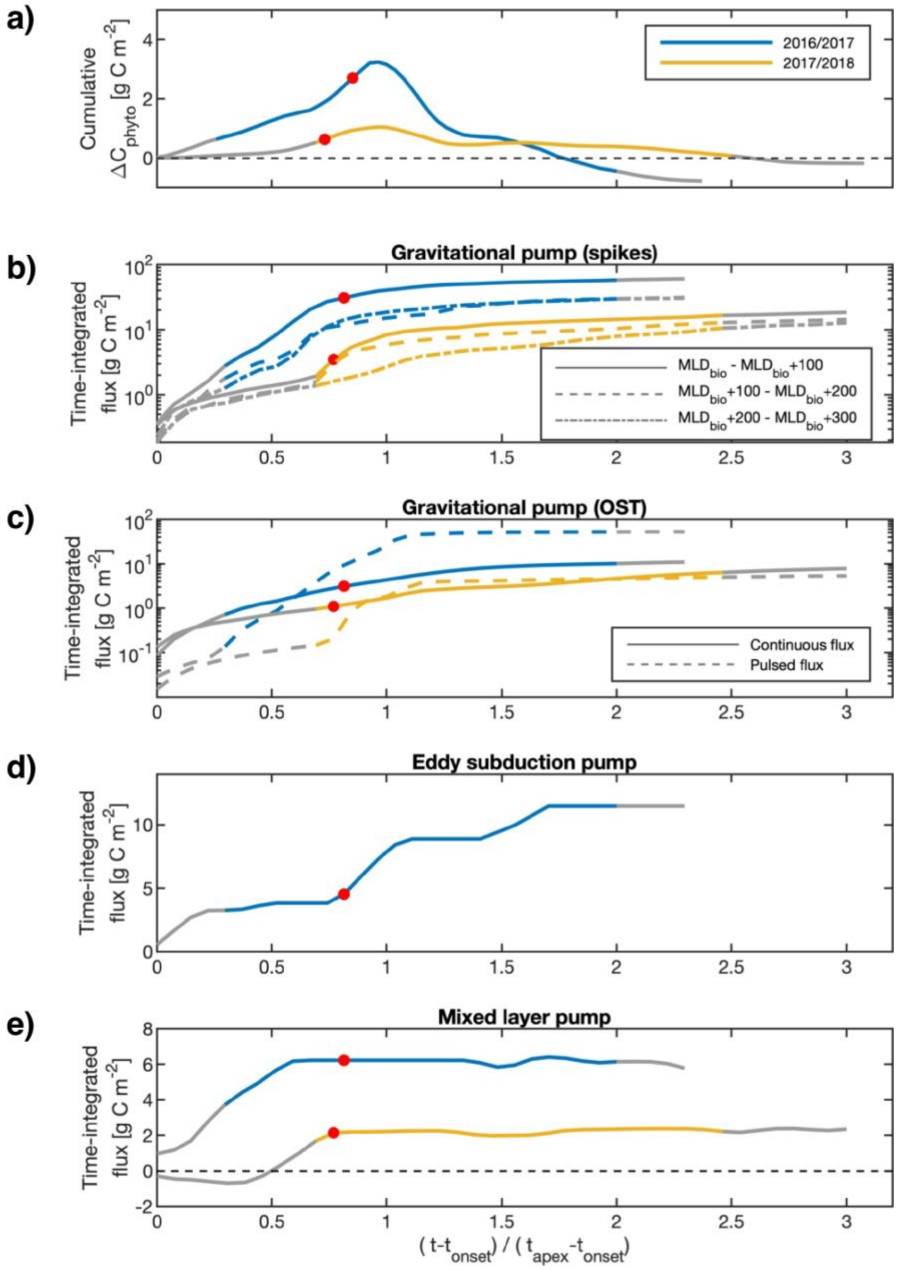

Figure S13 Relationship between phytoplankton phenology and carbon export pathways. a) cumulative sum of ΔC_{phyto}, the time increment of phytoplankton carbon. b) Time-integrated large-particle flux in three 100 m bins below the mixing layer depth estimated from large-particle backscattering b_{bl}. c) Time-integrated continuous (solid lines) and pulsed flux (dashed lines) estimated from optical sediment trap (OST) measurements. d) Time-integrated flux driven by the eddy-subduction pump (ESP) and e) the mixed layer pump (MLP). Each colour represents a different seasonal cycle. The coloured part of the curves indicates the region where we assumed a quasi-Lagrangian framework (See Fig. S2 and Methods). Red dots mark the timing of the bloom climax. The time axis was rescaled by the onset (t_{onset}) and the apex (t_{apex}) of the bloom, so that 0 corresponds to the onset and 1 to the apex. The end of the integration period of the seasonal cycle n is the date of the bloom onset of the cycle n+1, therefore the integration period is approximately one year. Note that the last seasonal cycle (2018-19) is incomplete and thus not shown.

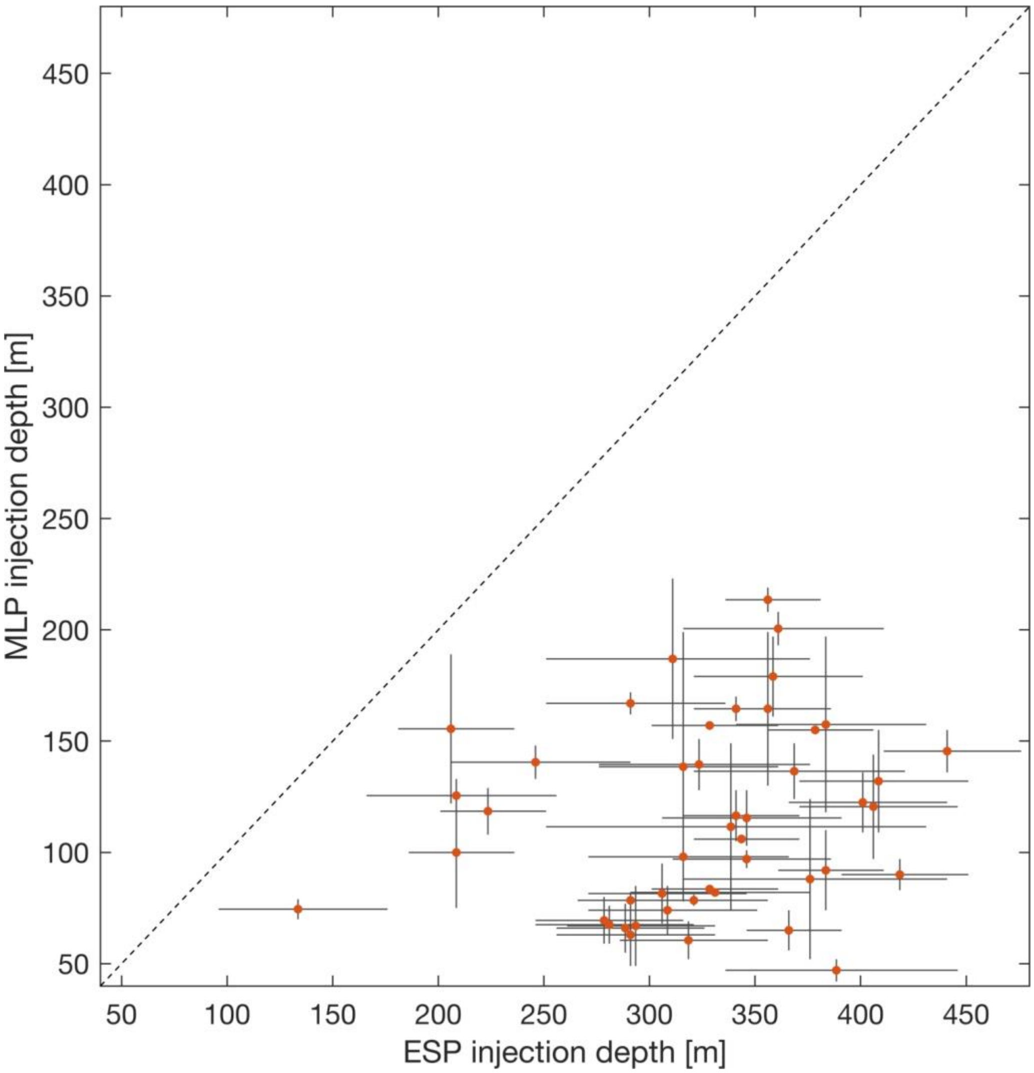

Figure S14 Mixed layer pump (MLP) versus eddy-subduction pump (ESP) injection depths. Vertical and horizontal bars represent the minimum and maximum depths of the remnant layers associated to the MLP and the subsurface features associated to the ESP, respectively.
